# An inflamed epidermal cyst treated with a vacuum-assisted biopsy device

**DOI:** 10.1093/jscr/rjag112

**Published:** 2026-02-27

**Authors:** Toshiyuki Yamaguchi

**Affiliations:** Department of Surgery, Division of Breast Diseases, Asama Nanroku Komoro Medical Center, 3-3-2-1, Aioicho, Komoro-shi, Nagano 384-8588, Japan

**Keywords:** inflamed epidermal cyst, vacuum-assisted biopsy device, reduction in inflammation

## Abstract

Inflamed epidermal cysts (ECs) are typically excised once inflammation subsides. Although incision and drainage are usually performed to reduce inflammation, we herein report a case of an inflamed EC, which was treated using a vacuum-assisted biopsy device (VABD). A 93-year-old woman presented with pain and redness in the left breast. We treated using VABD to achieve maximal lesion removal, and conducted a pathological examination of the obtained tissue. The lesion was pathologically diagnosed as an EC. Although the pain and inflammation had subsided within a week of treatment, a red nodule persisted. Because a remnant of the cyst wall was suspected, the lesion was excised and subsequently pathologically confirmed as a cyst remnant. While complete removal of an EC is challenging using VABD alone, the technique facilitates a reduction in inflammation.

## Introduction

Epidermal cysts (ECs) are one of the most commonly observed benign skin neoplasms [1, 2]. Although ECs are generally quiescent and asymptomatic, they can become red, swollen, and symptomatic, and are characterized by tenderness upon palpation and spontaneous pain [1, 2]. When an EC becomes symptomatic, it is referred to as an inflamed EC [3], and it is often difficult to differentiate it from malignancy or an abscess, despite detailed imaging [2]. As excision during active inflammation could be problematic, removal is generally postponed until inflammation subsides [2, 4–6]. Herein, we report a case of a patient with an inflamed EC who was treated using a vacuum-assisted biopsy device (VABD) to achieve maximal lesion removal and reduce inflammation.

## Case presentation

**Figure 1:**
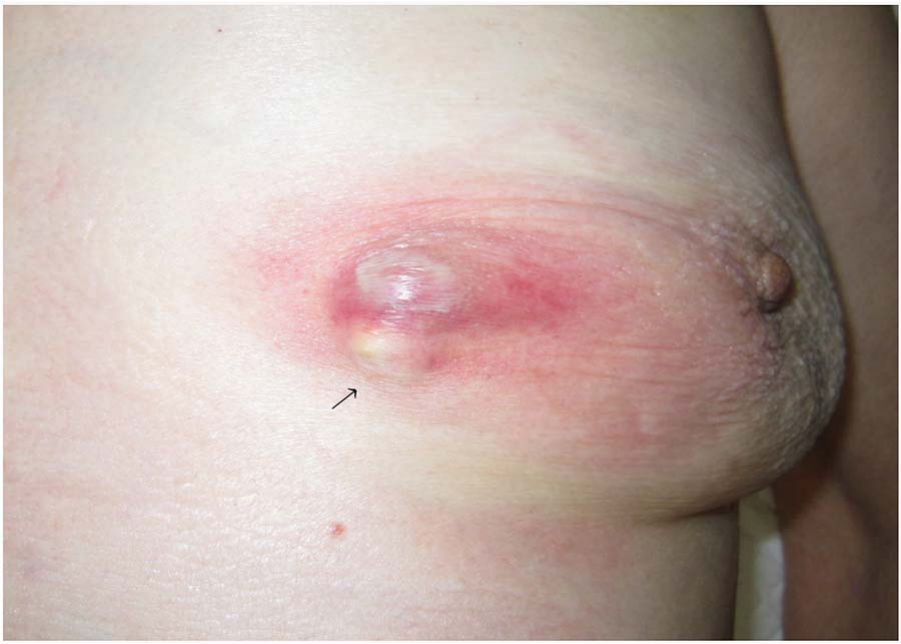
The left breast of a 93-year-old woman showing a nodule (arrow) of ~1 cm in diameter in the inner-lower region. This was accompanied by redness, blistering, and soreness of the surrounding skin.

**Figure 2:**
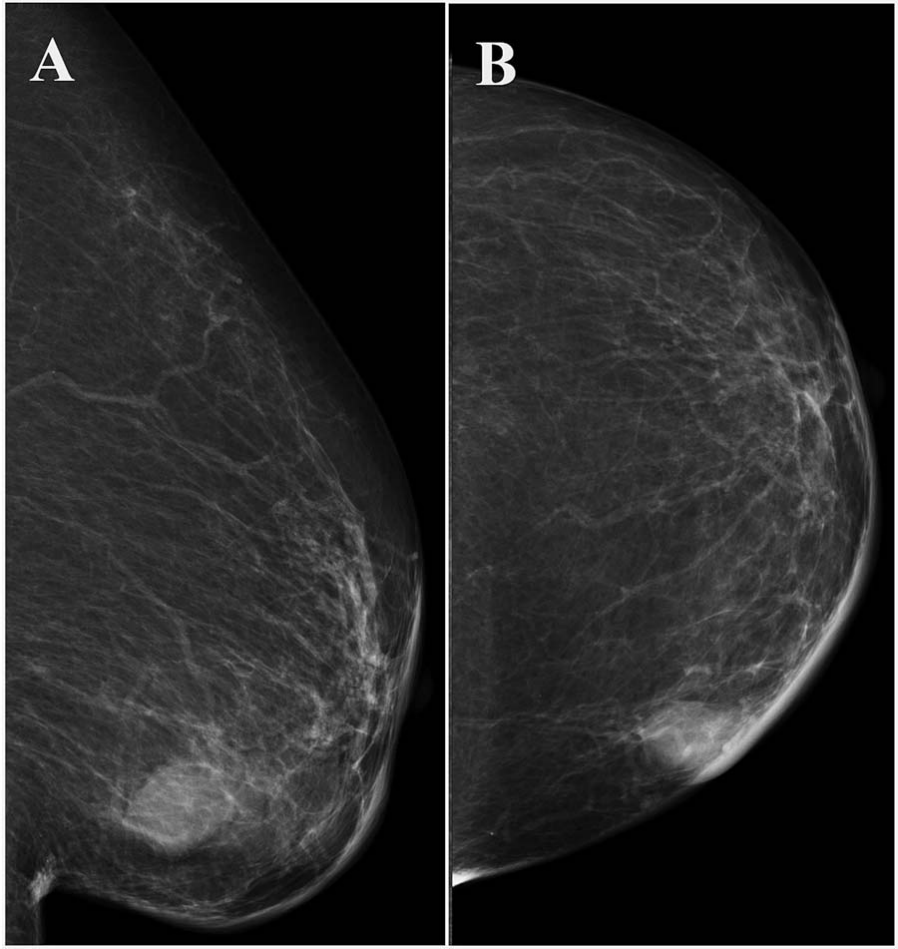
Left breast mammography showing a focal asymmetric density of ~1 cm in diameter in the inner-lower region. (A) Medio-lateral oblique view, (B) cranio-caudal view.

**Figure 3:**
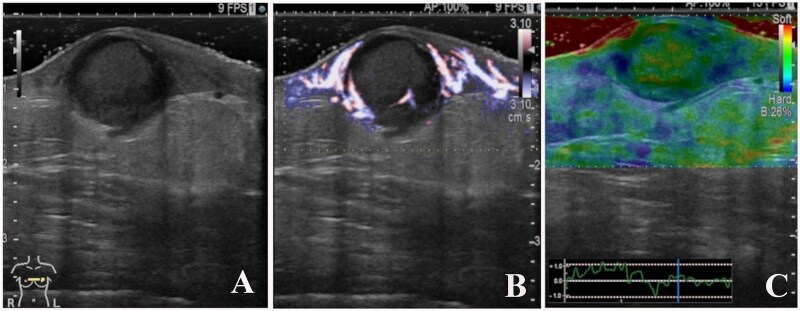
B-mode ultrasound imaging revealed that the nodule had an ill-defined, oval-shaped, and demonstrated dorsal acoustic amplification and lateral shadowing and heterogeneous pattern with a hypoechoic rim (A). Color doppler sonography showed blood flow signals mainly around the nodule (B), and on ultrasound elastography, the nodule appeared green-to-orange (C).

**Figure 4:**
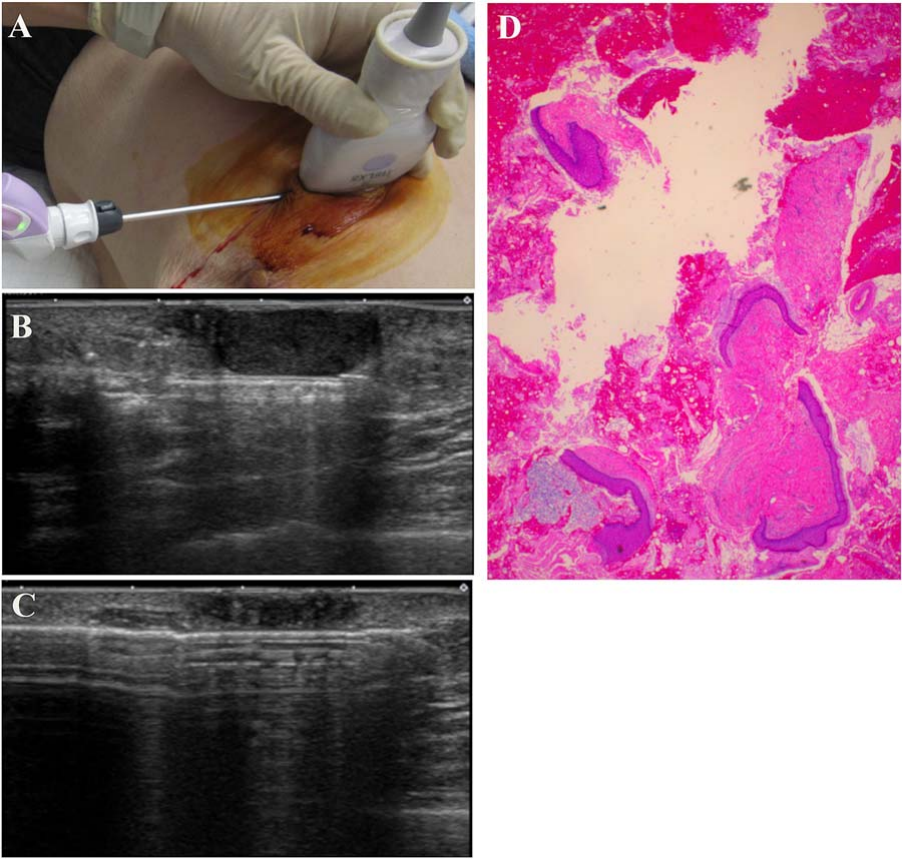
The operating needle was advanced through the skin incision (A) and positioned posterior to the lesion (B). The lesion was excised toward the skin using a rotating blade with vacuum aspiration (C). Histological examination revealed that the lesion comprised mature stratified squamous epithelium and laminated layers of keratin, which was consistent with the findings of an epidermal cyst (D, Hematoxylin and eosin stain).

**Figure 5:**
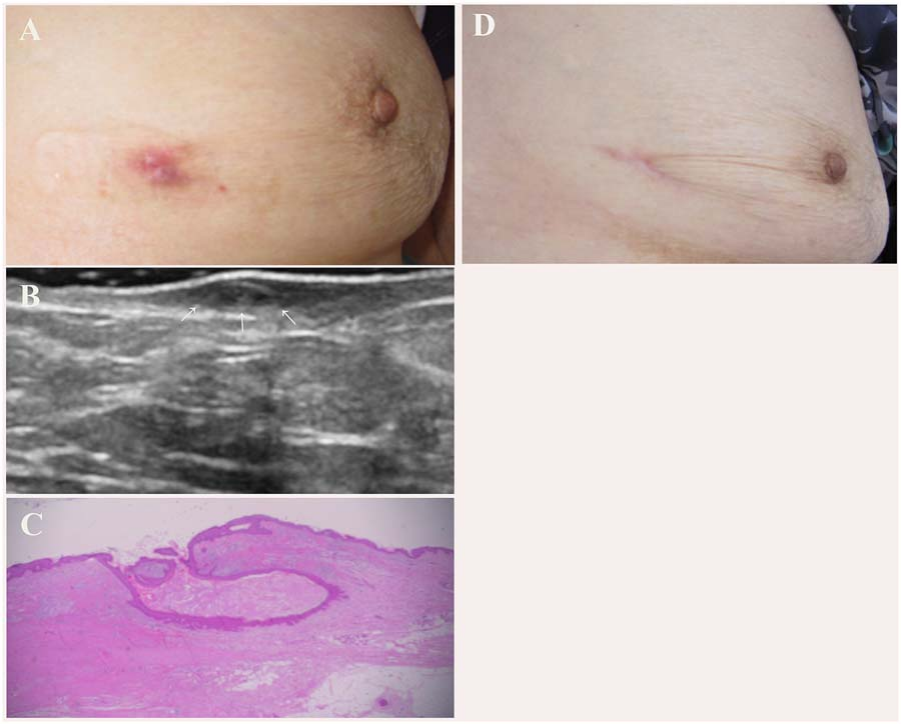
A red nodule of 4–5 mm in diameter remained following vacuum-assisted biopsy device treatment (A). B-mode ultrasound imaging showed skin thickening with a cystic lesion (arrow) (B). Pathologically, the lesion was identified as a remnant of the epidermal cyst (C, Hematoxylin and eosin stain). There was no evidence of recurrence at 3 years post-surgery (D).

A 93-year-old woman presented to the outpatient breast clinic with a 2-day history of pain and redness in the left breast. She reported that she had not noticed a nodule in left breast previously. Physical examination revealed a soft-to-hard nodule of ~1 cm in diameter in the inner lower region of the left breast, accompanied by redness, blistering, and soreness of the surrounding skin (Fig. 1). Her body temperature was 36.5°C, and laboratory data and vital signs were unremarkable. Mammography revealed a focal asymmetric density of ~1 cm in diameter in the inner lower region of the left breast (Fig. 2A and B), and B-mode ultrasound imaging indicated that the nodule was mainly located in the subcutaneous fat layer along with dorsal acoustic amplification and lateral shadowing, and was characterized by a heterogeneous pattern with a hypoechoic rim that was ill-defined on the nipple side (Fig. 3A). In addition, color Doppler sonography showed blood flow signals primarily near the nodule (Fig. 3B), whereas the nodule appeared green-to-orange on ultrasound elastography (Fig. 3C). We used an ultrasound-guided VABD (Mammotome ® Elite ™) for pathological examination and to reduce inflammation. After local anesthesia, a 5-mm skin incision was made away from the inflamed skin on the nipple side of the lesion. Under ultrasound guidance, a 10-gauge operating needle was advanced through the incision (Fig. 4A) and positioned posterior to the lesion (Fig. 4B). The direction of the needle head aperture was adjusted to face the lesion in multiple directions. The lesion was excised toward the skin using a rotating blade with vacuum aspiration (Fig. 4C), and this maneuver was repeated without significantly affecting the overlying skin. The time from insertion to removal of operating needle was 5 min, and the obtained specimens were sent for bacterial culture and pathological examination. Upon completion of the procedure, a compression dressing with gauze was applied for 1 day. No further wound healing care was provided. Histological examination revealed that the lesion comprised mature stratified squamous epithelium and laminated layers of keratin, which was consistent with an EC (Fig. 4D). Examination of bacterial cultures at 9 days after the treatment revealed the presence of *Prevotella bivia*, *Peptostreptococcus asaccharolytics,* and *Staphylococcus epidermidis.* However, the inflammation had subsided before the bacterial examination report was obtained. Although the symptoms and inflammation disappeared within a week post-treatment, a red nodule of 4–5 mm in diameter persisted (Fig. 5A). Ultrasound images

revealed thickened skin with a cystic lesion (Fig.  5B). Because we suspected it to be a remnant of the EC wall, excision of the lesion was recommended. Although the patient initially refused to provide consent, she subsequently agreed to undergo the procedure, and the lesion was excised 9 months after the VABD treatment. Pathologically, the lesion was identified as a remnant of an EC (Fig. 5C). At the time of writing of this case report, 3 years later, there has been no evidence of recurrence (Fig. 5D).

## Discussion

VABD is widely used for biopsy and treatment of breast lesions because of its less invasive nature and minimal, inconspicuous scarring [7]. However, using the VABD is challenging to excise lesions that are closely appressed onto the skin [7]. While striving for a perfect excision can lead to skin lacerations, adopting a more cautious approach to prevent skin injury may lead to incomplete removal. The EC is in extensive contact with the overlying dermis without any intervening subcutaneous fat [8, 9]. Therefore, VABD carries the risk of incomplete EC removal and recurrence. Thus, in many cases, subsequent excision of residual lesion may be necessary to prevent recurrence [10]. However, secondary excision of residual lesions can be a mental and physical burden for patients. Because a single-stage radical operation is standard for non-inflammatory ECs [2, 11], clinicians should exercise caution and thoroughly explain the limitations and considerations of the VABD procedure to patients before treatment.

In contrast, an inflamed EC is fragile and adheres to its surroundings [1]. Therefore, it is difficult to identify the dissection plane of an inflamed EC [1, 2, 12]. Consequently, excision of inflamed EC remains incomplete, resulting in frequent recurrence [1, 2, 6]. In general, inflamed ECs are treated in two stages rather than excision in a single procedure, and removal is planned after inflammation subsides [1, 6]. In this regard, incision and drainage (I & D) is widely recognized as an effective means of reducing inflammation in inflamed ECs [3–6]. In this procedure, the overlying skin is initially incised, and most of the cyst contents are evacuated and drained to reduce inflammation [1, 6]. Thereafter, removal of residual capsule is planned to prevent recurrence [1]. However, although effective, I & D, which involves packing, dressing change, and home care [4], tends to be inconvenient from a patient’s perspective [13, 14].

In the present case, we used VABD instead of I & D to treat inflamed EC. VABD can help reduce inflammation in ECs. Although, similar to I & D, removal of residual capsule is still required to prevent recurrence after inflammation subsides, compared with I & D, VABD may reduce patient’s inconvenience after treatment.
